# The relationship between type of drug therapy and blood glucose self-monitoring test strips claimed by beneficiaries of the Seniors' Pharmacare Program in Nova Scotia, Canada

**DOI:** 10.1186/1472-6963-8-111

**Published:** 2008-05-24

**Authors:** Chiranjeev Sanyal, Stephen D Graham, Charmaine Cooke, Ingrid Sketris, Dawn M Frail, Gordon Flowerdew

**Affiliations:** 1 Department of Community Health & Epidemiology, Faculty of Medicine, Dalhousie University, Halifax, Nova Scotia, Canada; 2 College of Pharmacy, Dalhousie University, Halifax, Nova Scotia, Canada; 3 Population Health Research Unit, Dalhousie University, Halifax, Nova Scotia, Canada; 4 Nova Scotia Department of Health, Halifax, Nova Scotia, Canada

## Abstract

**Background:**

The healthcare expenditure on self-monitoring of blood glucose (SMBG) test strips under the Nova Scotia Seniors' Pharmacare Program (NSSPP) has increased significantly in recent years. The objective of this study was to identify the frequency and cost of claims for blood glucose monitoring test strips by NSSPP beneficiaries in the fiscal year 2005/06 and to explore the variation in the use of test strips by type of treatment, age and sex.

**Methods:**

Retrospective analysis was conducted using pharmacy administrative claims data for NSSPP beneficiaries. Study subjects were aged ≥ 65 years on October 1, 2004, received SMBG test strips in the 110 days prior to April 1, 2005, and were alive throughout the twelve month study period. Subjects were categorized into four groups: insulin only, oral antihyperglycemic agents (OAA) only, both OAA and insulin; and no reimbursed diabetes medications. Statistical analysis was performed to identify differences in expenditure by medication group and in frequency of SMBG test strips claimed by medication group, age, and sex.

**Results:**

Of 13,564 included beneficiaries, 13.2% were categorized as insulin only, 53.5% OAA only, 7.2% both OAA and insulin, and 26.0% no reimbursed diabetes medications. Over half (58.7%) were femle. The insulin only category had the highest mean (± SD) number of SMBG test strips claimed per day (2.0 ± 1.5) with a mean annual total cost of $615 ± $441/beneficiary. Beneficiaries aged 80 years and above claimed fewer test strips than beneficiaries below 80 years.

**Conclusion:**

This population based study shows that in Nova Scotia the SMBG test strips claimed by the majority of seniors were within Canadian guidelines. However, a small proportion of beneficiaries claimed for SMBG test strips infrequently or too frequently, which suggests areas for improvement. The provincial drug plan covers the majority of the costs of test strip utilization, suggesting that the majority of test strips claimed did not exceed the maximum allowable cost (MAC) established in the program's MAC policy. Drug insurance programs need to work with healthcare providers to determine if patients are using test strips optimally; and to determine their impact on patient outcomes. In addition, they need to determine the cost-effectiveness of their SMBG test strip reimbursement policies.

## Background

Diabetes mellitus is an important public health issue [1]. The increasing prevalence, extensive morbidity, and associated high health care expenditures of diabetes pose an increasing burden on affected individuals, the health care system, and society as a whole [1]. Nova Scotia has one of the highest prevalences of diabetes in Canada [2] as approximately five percent of the population aged 20 years and above and fourteen percent of the population aged 65 years and above have diabetes [3]. With the increasing trend in diabetes prevalence, the total healthcare cost for individuals with diabetes in Canada is expected to increase by 75 % from $4.7 billion in 2000 to more than $8.1 billion in 2016[4]. The direct healthcare cost associated with diabetes in Nova Scotia is projected to increase from $175.5 million in 2005 to $249.3 million in 2016 [4].

Diabetes testing supplies are integral to SMBG which contributes substantial costs to the provincial health care plans. The provincial government drug benefit programs of Nova Scotia spent $2.66 million on insulin products, $4.10 million on oral antihyperglycemic agents (OAA) and $6.72 million on self-monitoring of blood glucose (SMBG) test strips in the fiscal year 2004/05 [Personal communication by Jianxiang Huang, M.D., Information and Statistics Officer, Nova Scotia Department of Health and Mike Joyce, BA., MBA, Senior Economic Advisor, Pharmaceutical Services, Nova Scotia Department of Health, March 19, 2008].

In the province of Nova Scotia, Canada, the Seniors' Pharmacare program (NSSPP) is a publicly funded drug insurance plan that reimburses drugs and medical supplies listed in the Nova Scotia Formulary for eligible seniors in the province. The beneficiaries of this program are Nova Scotia residents with a minimum age of 65 years who enroll by paying the required insurance premium and who pay the required per prescription co-payment. The NSSPP does not provide coverage for senior residents who have private drug insurance, or coverage with another public sector plan, e.g. with Veterans Affairs Canada or with First Nations and Inuit Health. The beneficiary co-payment during the study period was 33% of the total prescription cost to a maximum of $30 with a minimum of $3 per prescription. The annual maximum co-payment was $360. Once beneficiaries reached a total annual co-payment of $360, the plan covered 100% of eligible prescription costs. Co-payment is calculated according to the government fiscal year [5]. According to the NSSPP's cost sharing policy, a maximum allowable cost (MAC) of Canadian $0.74 was reimbursed for each test strip in the fiscal year 2004/05. Patients who wish to use test strips which cost more than the MAC, are required to pay the difference [5]. This cost sharing policy is intended to both reduce financial barriers to the practice of SMBG, while encouraging cost-effective SMBG through beneficiary contribution for the more expensive test strip brands.

Diabetes is associated with many complications, and inappropriate management can increase the risk of cardiovascular disorders, neurological disorders, foot sores and wounds, retinopathy, and nephropathy. Reducing hyperglycemia has shown to be an important factor in the prevention of diabetes-related complications [1]. Owing to the complex manifestations associated with diabetes, effective management of diabetes needs to be multifaceted with strategies which include: (1) educating patients about the signs and symptoms of the disease and how to assess their condition; (2) lifestyle interventions such as regular physical activity, appropriate diet, weight management, and reduced stress levels; and (3) medications when required [6,7]. Currently two methods, SMBG and laboratory monitoring of A1c are used to assess glycemic control in patients with diabetes. SMBG shows a patient's blood glucose levels at a particular time point whereas A1c shows a patient's blood glucose control in the past two or three months [8].

SMBG is considered to be a cornerstone of diabetes self-management to monitor blood glucose levels and guide patients in making adjustments to therapy or lifestyle to achieve glycemic control [1,8-12]. SMBG helps in avoiding episodes of high or low blood glucose levels among patients [13]. SMBG also allows physicians to gather data for appropriate clinical decision-making [14]. Qualitative studies have revealed patients' views on the advantages and disadvantages of SMBG. Patients report SMBG enhances awareness of lifestyle modifications needing adoption and raises the sense of success or failure to achieve target blood glucose levels [10,15]. Patients have also expressed the lack of interest shown by health professionals for the results of SMBG, compared to A1c levels, which discouraged them to continue with SMBG. This highlights the crucial role played by health professionals in educating patients on how to respond to testing results and in continuously reinforcing this information [10,15].

Furthermore, the frequency of SMBG has been found to vary among patients with diabetes in different jurisdictions [16-20]. An observational study evaluating insurance coverage policies for blood glucose devices and testing supplies found an increased rate of testing among patients once blood glucose monitors were provided free [21], while a randomized controlled trial (RCT) found no effect in 6-month glycosylated hemoglobin (A1c) between patients with Type 2 diabetes receiving free SMBG test strips and control subjects [22]. Patients with type 1 diabetes performing three or more self tests per day showed statistically and clinically significant association with A1c levels [23]. However the optimal frequency of SMBG per day is uncertain in patients with type 2 diabetes who are recommended to follow lifestyle interventions alone or in combination with OAAs [8,23-26].

Clinical practice guidelines recommend SMBG to both insulin and non insulin treated diabetes patients. The Canadian clinical practice guideline issued by the Canadian Diabetes Association in the year 2003 with a recommendation to test three or more times a day in patients receiving insulin and at least once a day in patients receiving oral antihyperglycemic agents [27]. These guidelines were synthesized by experts using standard criteria to assign levels of evidence from published studies.

The role of SMBG in patients with diabetes is widely debated [24,25,28]. Previous studies have proven the beneficial effect of SMBG in patients with type 1 diabetes [1,23] and in patients with type 2 diabetes who use insulin [8,23]. Patients with type 2 diabetes, who do not use insulin, but self monitor blood glucose, have not shown significant reduction in A1c levels in many prospective studies and randomized controlled trials [22,24,25,29-33] and a small effect of 0.39% decrease in A1c levels compared to control in one systematic review [30]. Studies assessing the effect of SMBG in non insulin treated patients have methodological issues, heterogeneity of the study population and variations in the interventions offered [34,35]. These studies lack homogeneity and the ability to measure the true impact of SMBG in non insulin treated patients [34,35]. Some experts support regular SMBG while others suggest that in non insulin treated patients, there is a need to weigh benefits with healthcare expenditures before advocating routine practice [34,35].

### Objective

The purpose of this population based study was to assess the utilization of SMBG test strips within a Canadian provincial drug plan which incorporates both a copayment and a policy of paying up to a maximum amount per test strip. We were interested in the pattern of test strips claimed by both insulin and non insulin treated patients and the extent of their adherence to guidelines for SMBG. The specific objectives of this study were (1) to analyze the frequency of SMBG test strips claimed by the beneficiaries of NSSPP based on treatment category, sex and age group; and (2) to estimate the government and beneficiary expenditure on SMBG test strips in the NSSPP, which has both a beneficiary copayment and a policy of paying up to a maximum amount per test strip.

## Methods

### Study Design

A retrospective database analysis was conducted using administrative pharmacy claims data to measure the frequency of SMBG test strips claimed, and healthcare expenditure on test strips.

### Study Population

The NSSPP program had 115,959 total beneficiaries in the fiscal year 2005/06. The NSSPP had 13,564 beneficiaries aged ≥ 65 years on October 1, 2004 who had received SMBG test strips in the 110 days prior to April 1, 2005 or at any time during the fiscal year 2005/06 and were eligible for Pharmacare for the entire period from October 1, 2004 to March 31, 2006. The 110 days time frame was chosen because the maximum days supply for each prescription in the NSSPP is 100 days.

Diabetes medications and SMBG test strips dispensed to beneficiaries were determined for the period October 1, 2004 to April 1, 2005 using World Health Organization's Anatomical Therapeutic Classification (ATC) [36] and Product Identification Number (PIN) assigned by the Pharmacy Association of Nova Scotia's (PANS) OPINIONS program [37]. Beneficiaries were classified into four groups depending on their pharmacy claims history:

1. Insulin products only,

2. OAA only,

3. Both insulin products and OAA,

4. No reimbursed diabetes medications.

The study population was stratified by sex (male, female) and age groups (65–69, 70–74, 75–79 and 80+ years) to assess variation in test strips claimed across these groups based on treatment. All the SMBG test strips dispensed to beneficiaries between April 1, 2005 and March 31, 2006 were included. The numbers of SMBG test strips dispensed were identified by PIN [37] from the pharmacy administrative claims data.

### Data Sources

Pharmacy administrative claims data were accessed through the Population Health Research Unit (PHRU), Dalhousie University [38]. To ensure confidentiality, patient records in the NSSPP database have unique encrypted patient identifiers according to Canadian Institute for Health Information (CIHI) encryption standards [38]. Pharmacy administrative claims data on SMBG test strips for the fiscal year 2004/05 and 2005/06 were examined. All data were provided to researchers at the individual patient level via a secure computing system. The Health Sciences Human Research Ethics Board, Dalhousie University, Halifax, Nova Scotia, approved this study.

### Data Analysis

The frequency of SMBG test strips claimed by NSSPP beneficiaries with diabetes in the fiscal year 2005/06 was estimated from the pharmacy claims data using descriptive statistics stratified by treatment group, sex, and age group. Univariate between-group comparisons were carried out to understand the relationship of age and sex with SMBG test strips claimed in the four treatment groups. The data had a skewed distribution hence the nonparametric Kruskal-Wallis test was used for between-group comparisons. The statistical significance level was set *a priori *at α = 0.05. The data analysis was conducted using SAS 9.1.3 [39]. Data are reported as mean ± standard deviation.

Healthcare expenditure on SMBG test strips claimed by beneficiaries in the fiscal year 2005/06 was estimated from the pharmacy claims. Between-groups comparisons were carried out using the same methods as the frequency analysis above. All results were reported in Canadian dollars.

## Results

### Characteristics of beneficiaries receiving SMBG test strips

13,564 beneficiaries of NSSPP were included in the study. More than one half of the beneficiaries were females (58.7%). The mean age of the beneficiaries was 74.9 years (standard deviation [SD]:6.7 years). There were 13.2% of beneficiaries identified to be receiving insulin products, 53.5% receiving OAA, 7.4 % receiving both insulin products and OAA, and the remaining 25.9 % had no claims for diabetes medications.

### SMBG test strips claimed by NSSPP beneficiaries

Table 1 shows the number of beneficiaries and mean number of SMBG test strips claimed per day per beneficiary by treatment group. Patients receiving insulin only or insulin with OAA claimed a mean [SD] of 2.0 [1.5] or 2.0 [1.3] test strips per day. Those taking OAAs without insulin claimed a mean [SD] of 1.0 [0.9] test strips per day, while those taking no diabetes medications claimed a mean [SD] of 0.7 [0.6] test strips per day. This variation by treatment group was statistically significant by the Kruskall-Wallis test (p < 0.001).

**Table 1:** Blood glucose monitoring test strips claimed and costs by treatment category, NSSPP for 2005/06

Treatment Category	N (%) Number of Beneficiaries	Mean (SD) SMBG Test Strips Claimed*	Mean (SD) Government Contribution Through the NSSPP^+ ^(Can $)	Mean (SD) Beneficiary Contribution Through Copayment and MAC policy^+ ^(Can $)	Mean (SD) Total Cost^+ ^(Can$)
Insulin products only	1786 (13.2)	2.0 (1.5)	562.17 (414.52)	52.34 (43.58)	614.50 (441.31)
Oral antihyperglycemic agents only	7252 (53.5)	1.0 (0.9)	271.61 (241.59)	39.90 (38.18)	311.51 (262.97)
Insulin products + Oral antihyperglycemic agents	1006 (7.4)	2.0 (1.3)	546.94 (361.59)	46.26 (38.90)	593.20 (382.50)
No reimbursed diabetes medications	3520 (25.9)	0.7 (0.6)	182.13 (177.88)	36.75 (34.84)	218.89 (197.77)

*Results per day

^+^Annual costs per beneficiary

There was no statistically significant variation in the number of test strips claimed by sex (p = 0.13). The variations by treatment category and by age were statistically significant (p < 0.001 and p < 0.02 respectively). The distribution of the numbers of SMBG test strips claimed by beneficiaries was highly (positively) skewed. Evidence of this is seen in Table 2.

**Table 2:** Blood glucose monitoring test strips claimed per day by treatment category, NSSPP for 2005/06

Treatment Category	Quartile 1 (Q1)	Quartile 2 (Q2)	Quartile 3 (Q3)	Quartile 4 (Q4)
Insulin products only	0.8	1.7	2.7	12.7
Oral antihyperglycemic agents only	0.3	0.8	1.4	9.9
Insulin products + Oral antihyperglycemic agents	1.1	1.6	2.7	8.5
No reimbursed diabetes medications	0.3	0.5	0.8	6.6

### Healthcare expenditure on SMBG test strips

NSSPP covered 91.5%, 87.2%, 92.2%, and 83.2% of the total cost for beneficiaries receiving insulin products only, OAA only, insulin products and OAA, and no reimbursed diabetes medications respectively. Table 1 shows the mean annual cost of SMBG test strips per beneficiary by treatment category.

The major proportion of the healthcare expenditure on SMBG test strips is covered by the government through the NSSPP because the MAC on test strips covers a significant proportion of the actual price of a number of brands of test strips. Also a large proportion of the beneficiaries likely reached their maximum annual copayment during the study period with the program subsequently paying the total amount. The difference in total cost by treatment category was statistically significant (p < 0.001). The use of private or third party insurance by the beneficiaries to cover their share was not known.

## Discussion

### SMBG test strips claimed by beneficiaries

This population-based study provides an insight into the patterns of SMBG test strips claimed by the beneficiaries of NSSPP in the fiscal year 2005/06. SMBG test strips claimed was used to identify the frequency of SMBG by the beneficiaries as in other studies [21]. However test strips claimed in our population by beneficiaries receiving OAA (about 30/month) greatly exceeded that in the study of diabetes patients using sulfonylureas in a United States (US) Health Maintenance Organization (approximately 10 test strips were used per month) [21]. The US study was conducted in 1995 when different clinical practice guidelines were in place [21]. In addition, Nova Scotia has a provincial diabetes care program with 39 centers across the province educating patients about the role of SMBG [40]. A study of the claims for SMBG test strips in the Drug Plan in Saskatchewan, Canada in 2001 noted that for patients with type 1 diabetes, the average patient's share of the cost was 50.9% compared to 8.5% in our study [12].

According to the Canadian Diabetes Association 2003 Clinical Practice Guidelines for the Prevention and Management of Diabetes in Canada, the optimal SMBG frequency for type 1 patients is three or more tests per day and at least one test a day for patients with type 2 diabetes [27]. In this study, the average daily SMBG test strips claimed for four treatment categories was: [1] 2 for insulin only; [2] 1 for OAA only; [3] 2 for insulin and OAA; and, [4] less than 1 for no reimbursed diabetes medications. The results suggest insulin users claim more test strips than non insulin users with the highest mean value for beneficiaries receiving only insulin therapy. These results suggest that current utilization in this population is in accordance with the Canadian clinical practice guidelines for patients receiving insulin alone or in combination with OAA [27]. There is lack of firm evidence to support daily self-monitoring for patients receiving only OAA [8,22-26,28,29]. Similarly there is limited evidence to support self-monitoring in those beneficiaries (>3000) who had no claims for antidiabetic medications. The lower frequency of in SMBG test strips claimed by beneficiaries aged ≥ 80 years was similar to previous studies which have found a decline in self-monitoring with increased age [20,41].

Table 2 shows a significant variation in SMBG test strips claimed by the beneficiaries in the fiscal year 2005/06. The highest and the lowest quartile results indicate extreme deviation from clinical practice guidelines. The lower and upper quartile values clearly suggest lack of concordance between SMBG by the beneficiaries and the clinical practice guidelines which recommend ≥ 3 self-tests per day for type 1 diabetes and at least one self-test daily for type 2 diabetes treated with medications [27].

Beneficiaries in the no reimbursed diabetes medications category may have received products not captured in the Pharmacare claims system. This may be confirmed by recognizing that:

(1) Beneficiaries might have been receiving insulin or an OAA not covered by NSSPP such as insulin glargine (Lantus^®^) or determir (Levimer^®^) ; chlorpropamide (Diabinese^®^); glimipiride (Amaryl^®^); repaglinide (GlucoNorm^®^); nateglinide (Starlix^®^) [42]. Hence they paid out of pocket for these products or their physicians might have given them samples provided by pharmaceutical representatives. A client sample (n = 1342) from a regional Nova Scotia diabetes care centre for the 12 month period of January 1, 2006 – December 31, 2006 included 501 seniors (37.4%). Of these 501 seniors, only 16 (3.2%) were taking one of the medications not reimbursed by Pharmacare [Personal communication by Zlatko Karlovic, B.E., Data Manager, Diabetes Care Program of Nova Scotia and Peggy Dunbar, MEd, PDt, CDE, Coordinator, Diabetes Care Program of Nova Scotia, March 31, 2008].

(2) Certain insulin and OAA products are covered under exception status for the NSSPP and beneficiaries must meet specific criteria to be eligible for coverage by NSSPP. Products for which coverage is contingent on meeting exception criteria include insulin lispro (Humalog^®^); insulin aspart (NovoRapid^®^); metformin/rosiglitazone (Avandamet^®^); rosiglitazone (Avandia^®^); pioglitazone (Actos^®^) [42].

(3) Some beneficiaries receive insulin injections from outpatient clinics which supply the medications and therefore no claims are made to the NSSPP.

(4) Beneficiaries may not initially fill a prescription for a diabetes medication until they determine whether they can stabilize their diabetes with diet and lifestyle modifications. A proportion of these beneficiaries may go on to fill a prescription for a diabetes medication many months after they start using SMBG test strips. However, a report examining a sample of patients registered in the Diabetes Care Program of Nova Scotia over age 19 in the calendar year 2006 indicated that of 6258 patients with type 2 diabetes, 1505 (24%) were managed by diet alone and 1137 (75.5%) of these patients reported SMBG. [Personal communication by Peggy Dunbar, MEd, PDt, CDE, Coordinator, Diabetes Care Program of Nova Scotia, January 16, 2008]

### Healthcare expenditure on SMBG test strips

This study highlights the healthcare expenditure on test strips for the fiscal year 2005/06 for NSSPP beneficiaries. The cost varied significantly among four treatment groups and was considerably higher for beneficiaries using insulin. The mean total cost of test strips per beneficiary was highest for beneficiaries only on insulin therapy. These results are similar to those reported in previous studies [12,43]. Despite the NSSPP's beneficiary copayment and its policy of paying only up to a maximum amount per test strip, the government covers the majority of the cost for test strips and a small portion is managed by the beneficiaries as shown in Table 1.

The increased claims and the associated costs of SMBG test strips for beneficiaries receiving insulin could be due to increased risk of hypoglycemia arising from insulin use and use of results to regulate insulin regimen [43]. Appropriate management of hypoglycemia through self testing has shown benefit in patients with type 1 diabetes [27,44]. However, evidence supporting the benefit of testing remains uncertain in non insulin treated patients [8,22,25,29,31]. In this study, the healthcare expenditure on self-monitoring by non insulin treated beneficiaries is significant for both the drug insurance plan and the individual beneficiary with lack of firm evidence supporting impact on long term outcomes and the cost effectiveness of such practice [22,35,45].

### Limitations

This study provides insight into use of SMBG test strips in a provincial pharmacare program of 115, 959 senior beneficiaries which covers 32 different brands of test strips.

Limitations to the study include: (1) test strips claimed by beneficiaries may not all have been used and we were unable to measure actual use and appropriateness of technique. (2) Test strips used by beneficiaries in hospitals were not determined. (3) Beneficiaries who have diabetes but do not practice SMBG and who did not receive test strips in the study period are not included. Beneficiaries who did not receive test strips for the entire study period might influence the study findings. (4) Changes in disease severity of beneficiaries during the study period who were later put on diabetes medications and practiced SMBG have not been included in the study. (5) We did not have actual diagnosis (type 1 or type 2) and a small number of beneficiaries receiving only insulin may have type 2 diabetes. (6) A small percentage of beneficiaries who receive insulin (such as Lantus^®^) or OAA agents (certain thiazolinediones or sulfonylurea derivatives) which are not reimbursed by the NSSPP may have been assigned to an incorrect treatment group [42]. (7) The NSSPP does not cover citizens over 65 years of age who have drug coverage through a federal or private insurance program and those over 65 years who chose not to pay the Nova Scotia Seniors' Pharmacare premium; hence they were excluded in this study. (8) It is not known whether the beneficiaries made private insurance claims to cover their share of the cost.

## Conclusion

This population based study shows in the Canadian province of Nova Scotia, the SMBG test strips claimed for the majority of the senior beneficiaries were found to be within Canadian guideline recommendations. However, the results show that a small proportion of beneficiaries who claimed for SMBG test strips infrequently or too frequently which suggests areas for improvement. The provincial drug plan covers the majority of the costs of test strips utilization, suggesting the majority of test strips claimed do not exceed the maximum allowable cost (MAC) established in the program's MAC policy [5]. Drug insurance programs need to work with health care providers to determine if patients are using test strips optimally; and to determine their impact on patient outcomes. In addition, they need to determine the cost effectiveness of their SMBG test strip reimbursement policies.

## Abbreviations

A1c: Glycosylated hemoglobin, ATC: Anatomical Therapeutic Classification, CIHI: Canadian Institute for Health Information, MAC: Maximum allowable cost, NSSPP: Nova Scotia Seniors' Pharmacare Program, OAA: Oral Antihyperglycemic Agents, PHRU: Population Health Research Unit, PIN: Product Identification Number, RCT: Randomized control trial, SD: Standard deviation, SMBG: Self-monitoring of blood glucose.

## Competing interests

Charmaine Cooke and Chiranjeev Sanyal received salary support from an unconditional grant from the Nova Scotia Department of Health to Dalhousie University. Dr. Ingrid Sketris has received a grant from the Nova Scotia Department of Health.

## Authors' contributions

CS was involved with study design, data analysis, disseminating results and preparing the manuscript. SDG designed the study, planned the analysis, coordinated the project and critically revised the manuscript. CC was involved with study design, coordinating the project and critically revising the manuscript. IS participated in the design of the study, coordinated the project and critically revised the manuscript. DMF conceived the project and brought in policy perspectives to the project. GF contributed statistical expertise and critically revised the manuscript. All authors have read and approved the final version.

## Pre-publication history

The pre-publication history for this paper can be accessed here:


